# Do marine reserves increase prey for California sea lions and Pacific harbor seals?

**DOI:** 10.1371/journal.pone.0218651

**Published:** 2019-06-20

**Authors:** Alejandro Arias-Del-Razo, Yolanda Schramm, Gisela Heckel, Andrea Sáenz-Arroyo, Arturo Hernández, Leonardo Vázquez, Aldo I. Carrillo-Muñoz

**Affiliations:** 1 Departamento de Ciencias Químico-Biológicas, Universidad de las Américas Puebla, San Andrés Cholula, Puebla, Mexico; 2 Facultad de Ciencias Marinas, Universidad Autónoma de Baja California, Ensenada, Baja California, Mexico; 3 Departamento de Biología de la Conservación, Centro de Investigación Científica y de Educación Superior de Ensenada, Ensenada, Baja California, Mexico; 4 El Colegio de la Frontera Sur, Unidad San Cristóbal, San Cristóbal de Las Casas, Chiapas, Mexico; 5 Comunidad y Biodiversidad A.C., Guaymas, Sonora, Mexico; 6 Centro Tlaxcala de Biología de la Conducta, Universidad Autónoma de Tlaxcala, Santa María Acuitlapilco, Tlaxcala; Institute of Marine Research, NORWAY

## Abstract

Community marine reserves are geographical areas closed to fishing activities, implemented and enforced by the same fishermen that fish around them. Their main objective is to recover commercial stocks of fish and invertebrates. While marine reserves have proven successful in many parts of the world, their success near important marine predator colonies, such as the California sea lion (*Zalophus californianus*) and the Pacific harbor seal (*Phoca vitulina richardii*), is yet to be analyzed. In response to the concerns expressed by local fishermen about the impact of the presence of pinnipeds on their communities’ marine reserves, we conducted underwater surveys around four islands in the Pacific west of the Baja California Peninsula: two without reserves (Todos Santos and San Roque); one with a recently established reserve (San Jeronimo); and, a fourth with reserves established eight years ago (Natividad). All these islands are subject to similar rates of exploitation by fishing cooperatives with exclusive rights. We estimated fish biomass and biodiversity in the seas around the islands, applying filters for potential California sea lion and harbor seal prey using known species from the literature. Generalized linear mixed models revealed that the age of the reserve has a significant positive effect on fish biomass, while the site (inside or outside of the reserve) did not, with a similar result found for the biomass of the prey of the California sea lion. Fish biodiversity was also higher around Natividad Island, while invertebrate biodiversity was higher around San Roque. These findings indicate that marine reserves increase overall fish diversity and biomass, despite the presence of top predators, even increasing the numbers of their potential prey. Community marine reserves may help to improve the resilience of marine mammals to climate-driven phenomena and maintain a healthy marine ecosystem for the benefit of both pinnipeds and fishermen.

## Introduction

Marine reserves are geographic areas designated for the conservation and management of ecosystems and natural resources [1]. While different management strategies may be applied according to each reserve’s goals, community marine reserves usually consist in areas where no extraction of marine species is allowed. Proliferating around the world as an efficient and inexpensive way to maintain and manage fisheries while also preserving biodiversity [2], they have proven successful in increasing fish biomass, organism size and diversity within their confines [2]. They have also been found to export recruits beyond their borders, even on a regional scale [3], and have been proven to increase ecosystem resilience to harmful conditions, such as climate driven hypoxia [4].

**Fig 1:**
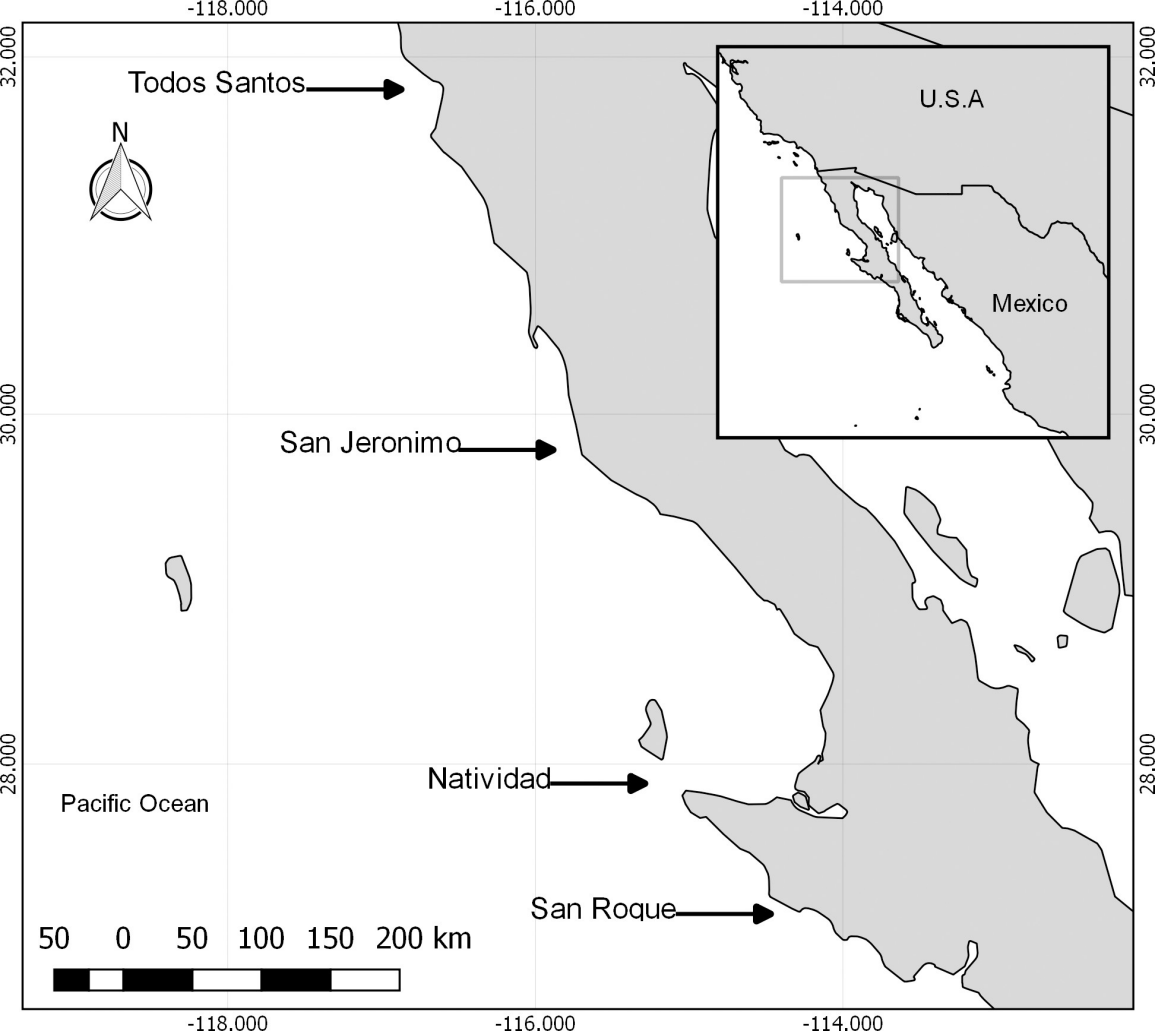
Study area. The study area comprised four islands in the Pacific Ocean off the west coast of the Baja California Peninsula, Mexico.

Most islands in the Pacific Ocean off the west coast of the Baja California Peninsula, Mexico, have important populations of pinnipeds, mainly California sea lions (*Zalophus californianus*) and Pacific harbor seals (*Phoca vitulina richardii*), but also northern elephant seals (*Mirounga angustirostris*) and Guadalupe fur seals (*Arctocephalus philippii townsendi*) [5]. Local fishing cooperatives have exclusive rights to the marine resources around these islands, granted by an official concession from the Mexican government. Their main fisheries are lobster (*Panulirus interruptus*) and abalone (*Haliotis fulgens* and *H*. *corrugata*) [6], with cooperative fisheries with exclusive rights exploiting these species around all four islands surveyed in the present study (Fig 1). The number of fishermen, target species and fishing gear pertaining to the cooperatives on San Jeronimo, Natividad and San Roque are similar [7]. On Todos Santos, which is also close to a major urban center and fishing port [7], an aquaculture company operates fish enclosures in the southeast portion of the southernmost of these two islands.

**Fig 2:**
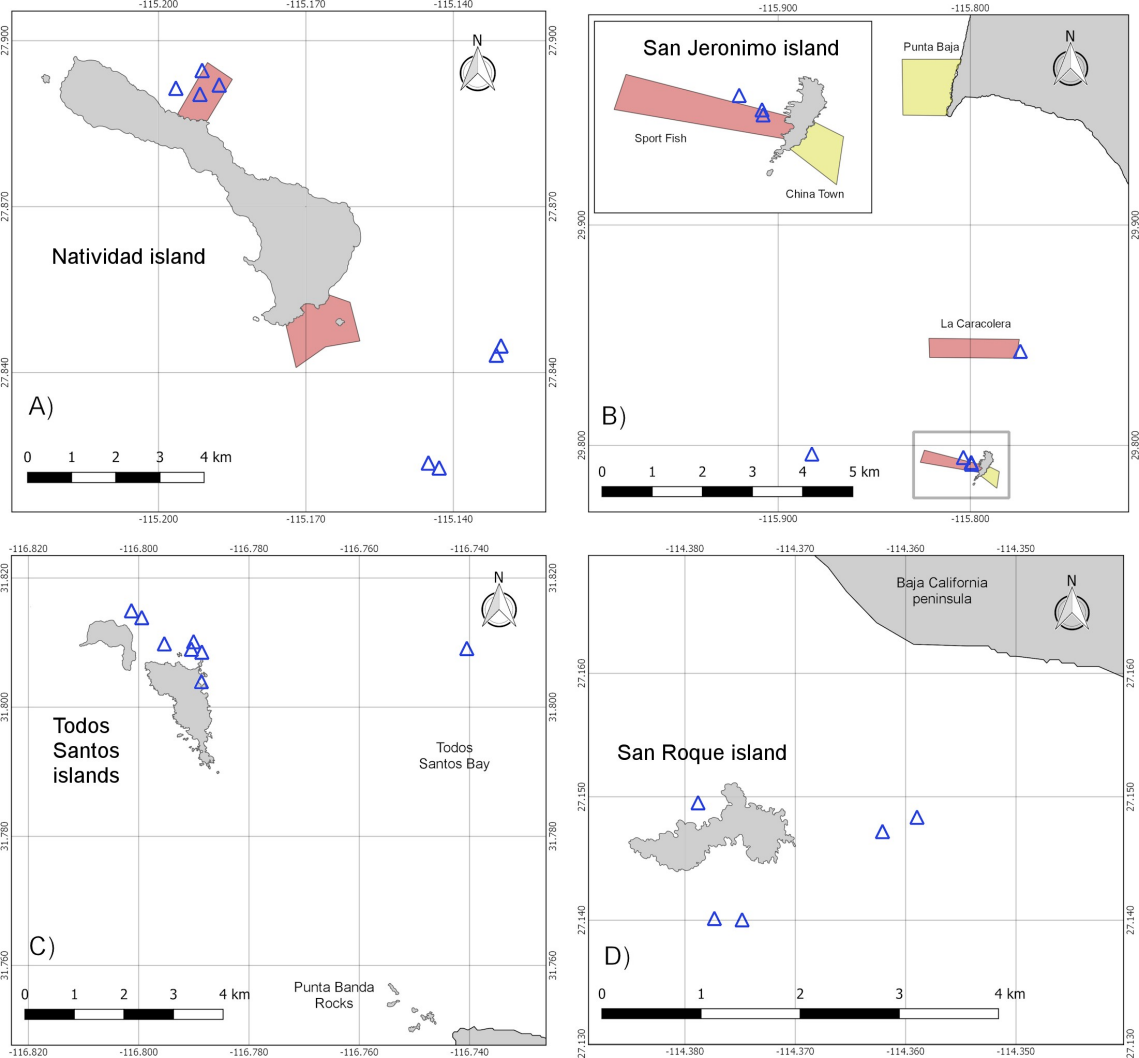
Survey cylinders and community marine reserves. The blue triangles represent survey cylinders, yellow polygons represent partially closed reserves and red polygons fully closed reserves. **A) Natividad island.** It has two marine reserves, with a total extraction restriction implemented in 2006. **B) San Jeronimo Island**. There are four reserves near the island, established in 2014. **C) Todos Santos islands.** Although the local fishing cooperative has exclusive rights to the waters close to the islands’ shores, an aquaculture company also operates to the southeast of Todos Santos South. Industrial fishing is also carried out in the waters of the bay, with a commercial and fishing port nearby. **D) San Roque Island.** There are only harbor seals on this island. However, there is a significant sea lion colony on nearby Asuncion Island, 9.5 km to the southeast.

The abundance of abalone has declined over the last five decades, with recent total catches six times lower than those registered throughout the 1960s and 70s and ten times lower than record catches from the early 1950s [8]. This has led some fishing cooperatives to look for a way to recover abalone populations. In 2006, the fishing cooperative *Buzos y Pescadores de la Baja California*, *S*.*C*.*L*. (Divers and Fishermen of Baja California), with the advice of the non-governmental organization *Comunidad y Biodiversidad A*.*C*. (Community and Biodiversity) (COBI), established two marine reserves around Natividad Island (Fig 2A). The main goal was to protect the ecosystem in which abalone lives, in order to maintain the productivity of *Haliotis fulgens* and *H*. *corrugata* [9]. The largest of the two reserves, *La Plana*, covers 1.3 km^2^, while the second, *Punta Prieta*, comprises 0.7 km^2^ (Fig 2A) [10].

Since the establishment of the marine reserves, another four were established, in 2014, around San Jeronimo Island by COBI and the fishing cooperative *Ensenada S*.*R*.*L* (Fig 2B). Two of the reserves, *Sport Fish* and *China Town*, are adjacent to the island, with the third, *La Caracolera*, found between the island and the peninsula, and the last adjacent to the mainland, west of Punta Baja (Fig 2B). Two of the reserves are partially closed, while the other two are totally closed to any kind of extraction. The main goals for establishing these reserves were to protect the habitat of the abalone, recover its populations, and increase fish recruitment. *Punta Baja* reserve covers an area of 7.15 km^2^, while *La Caracolera*, *Sport Fish* and *China Town* comprise 4.22 km^2^, 1.56 km^2^, and 0.55 km^2^, respectively (Fig 2B) [A. Hernandez *personal comm*. 2017].

One of the concerns the fishermen expressed during marine reserve planning meetings with biologists is that the implementation of these marine reserves would solely benefit the pinnipeds on the islands, especially the populations of Pacific harbor seals and California sea lions. They expected that the reserves would facilitate the growth of these populations, thus neutralizing their intended positive effect in terms of available resources for the fishermen. Similar concerns have been expressed for the marine reserves in Puget Sound, Washington, anticipating that harbor seals would be attracted to increased prey abundance in these areas [11]. In this region, the California sea lion’s diet is composed mainly of fish and cephalopods, although it may include crustaceans, especially during abnormal oceanographic conditions [12]. Harbor seals in this region prey mainly on benthic fish, such as California lizardfish (*Synodus lucioceps*) or flatfish (*Citharichthys* spp.), but also on squid (*Doryteuthis opalescens*) and octopuses (*Octopus* spp.) [13].

Few studies have focused on the possible effects of no-take marine reserves on top predators. A comparison between undisturbed and exploited coral reef communities found significantly higher top predator (such as sharks) biomass and density on undisturbed communities [14]. Research conducted in an experimental marine reserve next to a major African penguin (*Spheniscus demersus*) colony found that the foraging effort in the colony next to the reserve reduced by 25 to 30% compared with the year prior to the establishment of the reserve. Meanwhile, no significant difference in foraging effort between both years was found in another colony which did not have an established marine reserve [15]. However, the above described findings should not lead to the expectation that all species increase in abundance and biomass within marine reserves. Given that no take-reserves change the dynamics of the community, it should be expected that some species increase in abundance while others decline [16]. Red abalone (*Haliotis rufescens*) has shown lower densities in marine reserves where sea otters (*Enhydra lutris*) have been reintroduced [17]. In conclusion, these studies have shown that protected or undisturbed areas tend to favor top predators like harbor seals and other pinnipeds, which should be taken into account when planning marine reserves for the recovery of depressed stocks [11].

In light of the above, we hypothesized that the marine reserves established at *Natividad* and *San Jeronimo* islands may be effective in increasing overall fish biomass and diversity, despite the pinniped populations on the islands.

To assess the above described effect, we compared fish biomass and diversity around four islands off the west coast of the Baja California peninsula: two without marine reserves–Todos Santos and San Roque; San Jeronimo, with reserves established the same year as our surveys were conducted; and, Natividad, with reserves that had been established for eight years (since 2006) by the time our surveys were conducted in 2014. There are important harbor seal colonies at all of these sites, while, on Todos Santos, San Jeronimo and Natividad, California sea lion colonies are also found.

## Methods

We conducted underwater surveys around four islands along the Pacific coast of the Baja California peninsula, Todos Santos, San Jeronimo, Natividad and San Roque, with important sea lion and harbor seal rookeries [18] (Fig 1). Although there are marine reserves close to San Jeronimo and Natividad, the sampling sites chosen, both within these reserves and beyond, were over rocky substrates and in favorable visibility and wave strength conditions (Fig 2).

The surveys comprised the use of survey cylinders and were undertaken by divers from the local community, certified by COBI for scientific diving, and by scientific divers from the *Universidad Autónoma de Baja California* (UABC, or Autonomous University of Baja California). Each diver stood suspended on the water column at a depth of between 9 and 22 meters, and took a series of point-count snapshots of the fish present in the 360° around their position and at a distance of up to two meters, for 15 minutes [19]. The divers recorded location coordinates, date, depth, water temperature, species, number of individuals and size. This process was repeated between 11 and 16 times per island.

For macroinvertebrates, a different team of divers swam close to the bottom, at a depth of between 11 and 24 meters, following a 30-meter long transect (60m^2^) and recording all macroinvertebrates one meter to the right and left of the transect line [19], with between 14 to 16 repetitons per island. It should be noted that bad weather conditions forced the suspension of the survey on San Jeronimo Island after only five surveys. All surveys were conducted during August and September 2014.

### Fish biomass

Fish biomass was calculated using Froese and Thorson’s [20] Bayesian approach for estimating length-weight relationships in fish, with A and B parameters for the length-weight relationships obtained from FishBase [21].

To compare prey biomass, the potential prey species for pinnipeds were selected based on prior dietary studies conducted in this region on California sea lions [12] and harbor seals [13]. Invertebrate prey, such as squid and octopus, were excluded here because we did not have data on such species.

### Data analysis

In order to analyze the effect of the implementation of marine reserves on the biomass around these islands, we constructed different generalized linear mixed models (GLMM) [22]. Fish biomass was used as the response variable, while years of protection (the period of time elapsed from the establishment of the reserves to the sampling date) and protection status (whether the site was inside or outside a reserve) were used as explanatory variables. The sampling sites around the islands were used as the random factor. We constructed different models with different combinations of variables and selected the best according to its Akaike Information Criterion (AIC) score, with the lowest score indicating a more parsimonious model [23]. We used R (*A Language and Environment for Statistical Computing*) version 3.5.3 to construct the GLMMs [24].

The same analysis was employed using the biomass of potential California sea lion and Pacific harbor seal prey, but using a filter database of fish biomass constructed according to the known prey of each pinniped species [12, 13, 25, 26].

Species accumulation curves were used to measure fish and macroinvertebrate species richness, while Simpson’s diversity index, using EstimateS 9.1, measured biodiversity [27]. We chose the Simpson index because it tends to be more sensitive than the Shannon index to the dominant species in a community [28], thus complementing the species richness information provided by the species accumulation curve. We compared the fish diversity around each island via one-way ANOVA tests and *a posteriori* Tukey tests, while a Kruskal-Wallis test was applied to establish invertebrate diversity. Minitab 17 was used for both analyses [29].

## Results

### Fish biomass

**Fig 3:**
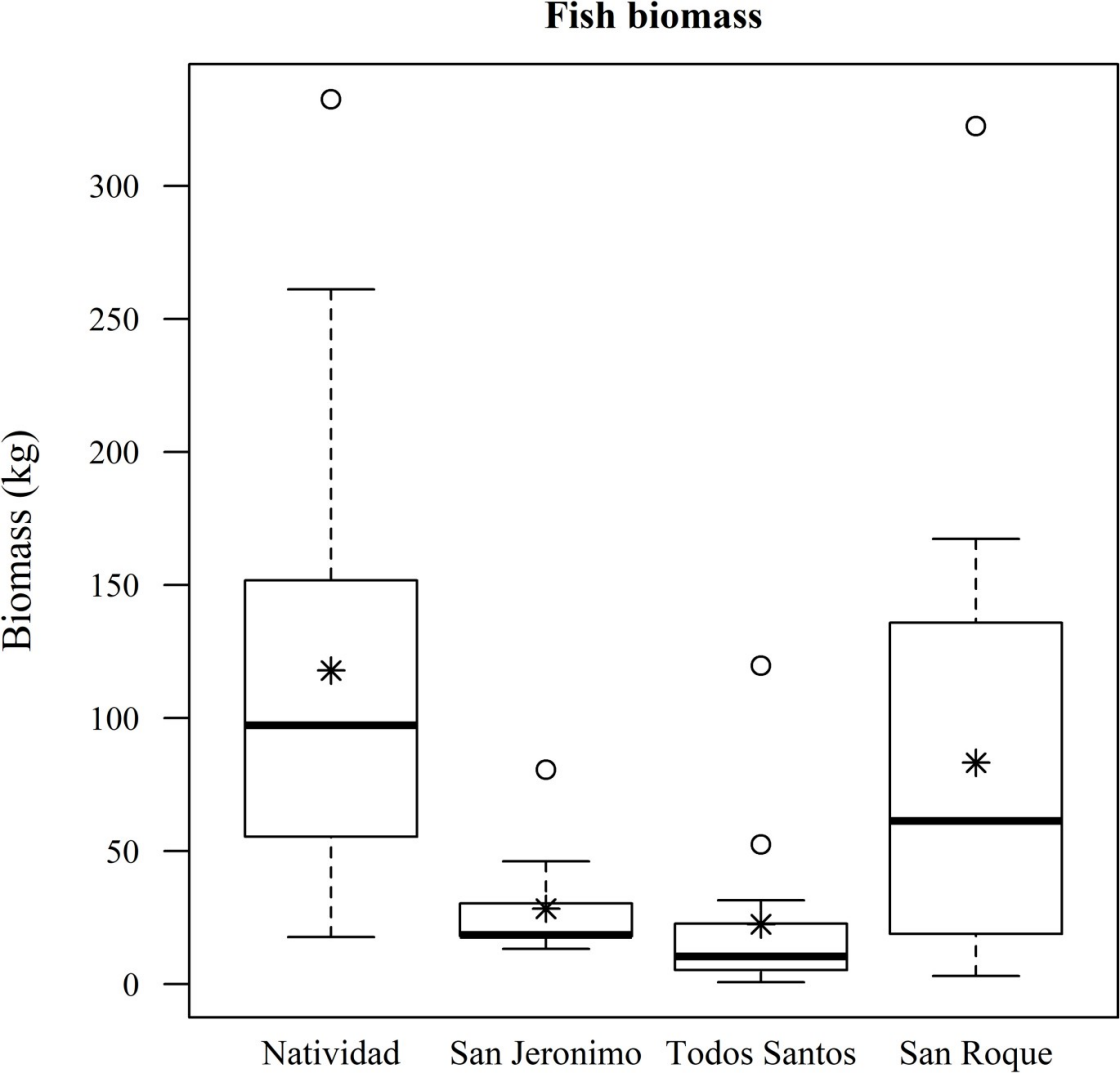
Fish biomass. The asterisks represent the mean fish biomass, the circles represent the outliers, the whiskers represent upper and lower quartiles, the boxes represent the interquartile range and the bands inside the boxes represent the median.

**Fig 4:**
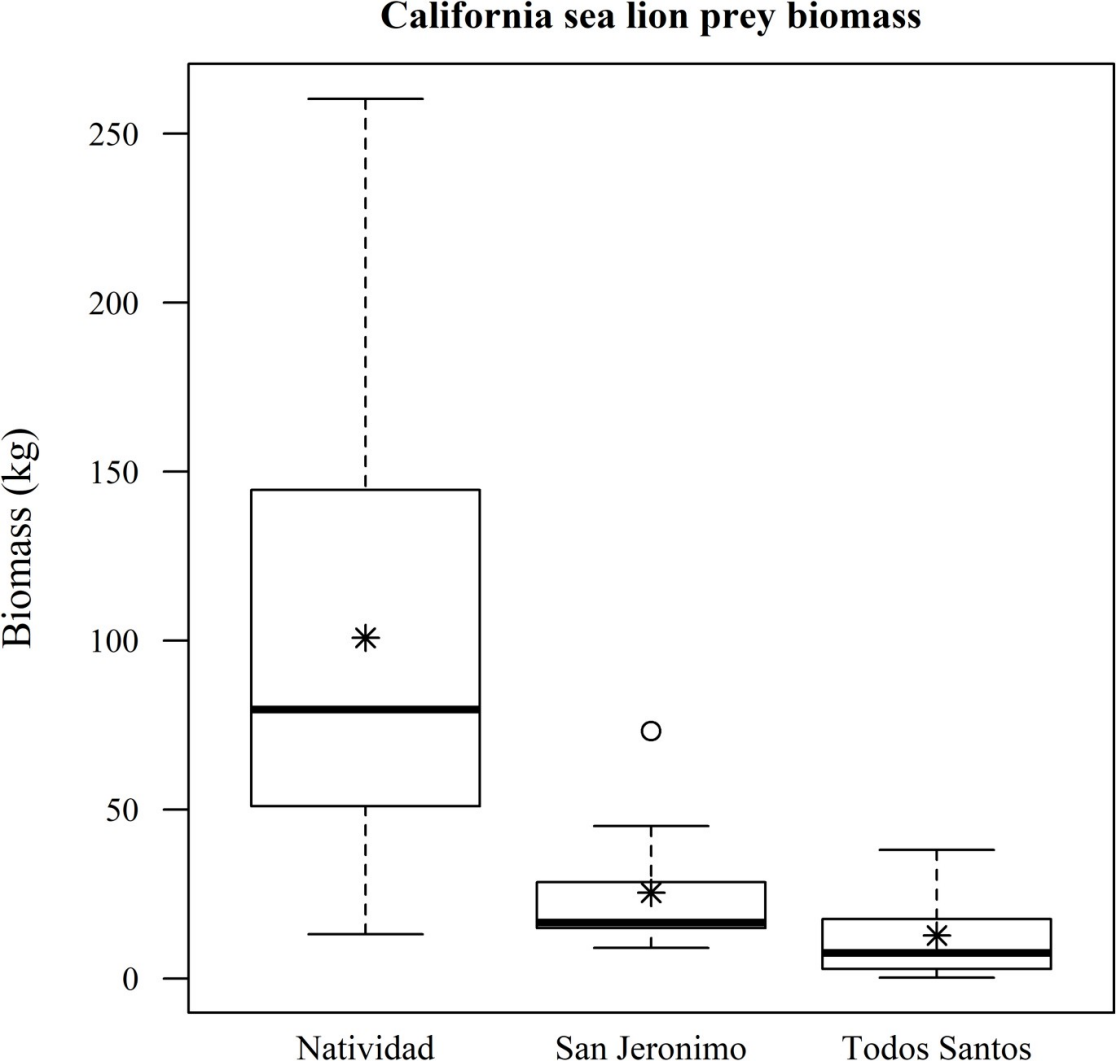
Fish biomass of potential California sea lion prey. Only the reported fish prey of California sea lions are included. The asterisks represent the mean fish biomass, the circles represent the outliers, the whiskers represent upper and lower quartiles, the boxes represent the interquartile range and the bands inside the boxes represent the median.

**Fig 5:**
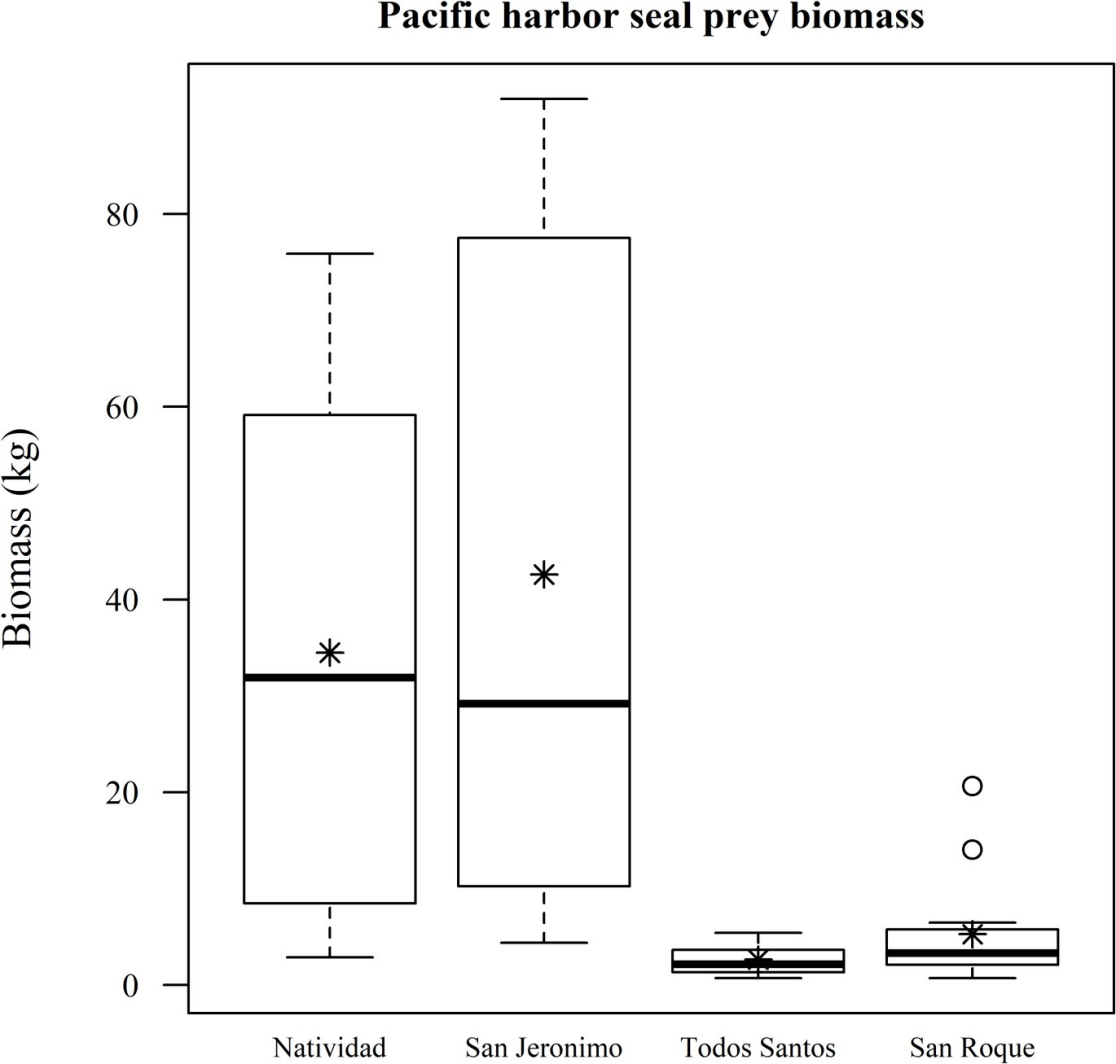
Fish biomass of potential Pacific harbor seal prey. Only the reported fish prey of Pacific harbor seals are included. The asterisks represent the mean fish biomass, the circles represent the outliers, the whiskers represent upper and lower quartiles, the boxes represent the interquartile range and the bands inside the boxes represent the median.

Fish biomass, considering all species and survey points both within marine reserves and beyond (n = 55), was highest around Natividad Island, followed by San Roque, San Jeronimo and Todos Santos islands (Fig 3). After filtering the results for known California sea lion fish prey, biomass was also found to be higher around Natividad than around Todos Santos and San Jerónimo (Fig 4). We did not include San Roque because there are no sea lion rookeries there. Both Natividad and San Jeronimo recorded similarly high mean numbers for the fish biomass of potential Pacific harbor seal prey, although the confidence intervals are very wide, while San Roque and Todos Santos presented very low biomass levels (Fig 5).

In all models, the data show a gamma distribution. According to its AIC value, the best model uses the fish biomass as the response variable and both the level of protection of the site (inside or outside of a reserve) and the years since the marine reserve were established as explanatory variables (S1 Table). The results show that the age of the marine reserve around the islands is a significant factor with a positive trend (p = 0.037), while the protection of the site (inside or outside of the reserve) was not (p = 0.694; S2 Table). This means that the biomass around Natividad Island, with marine reserves established eight years prior to the survey, tended to be higher than that found around San Jeronimo, protected for one year, or Todos Santos and San Roque, which lack this protection entirely. Additionally, the protection status of the sampling site had little effect on fish biomass levels, which tended to be similar both inside and outside the marine reserves. This could potentially mean that there is a spillover effect around the marine reserves. The model showed a significant intercept (p = 0.001), indicating that biomass around islands without a reserve is different from zero.

After filtering the biomass of potential California sea lion prey, the best model used potential prey biomass as the response variable, and the protection of the sampling site and the years since the establishment of the marine reserve as explanatory variables (Table 1). In this model, we eliminated the results from San Roque because there are no California sea lion rookeries on that island. The results show that the age of the marine reserves had a significant positive effect on prey biomass (p = 0.001), while the protection status of the site did not (p = 0.938). The intercept was significant (p = 0.001; Table 2). As with the general biomass, this result shows that islands with older marine reserves, such as Natividad, have a significantly higher prey biomass, which, in this research, was similar both inside and outside the reserves.

**Table 1:** GLMMs of the fish biomass of California sea lion prey against years and protection of the site.

Fixed effects	AIC	Deviance	Variance of Residuals
**Anything**	52528	52520	11.295
**Years of protection**	52522	52512	11.29
**Years and protection of the site**	52524	52512	11.29
**Protection of the site**	52530	52520	11.295
**Years, protection and interaction**	52526	52512	11.284

**Table 2:** GLMMs of the fish biomass of California sea lion prey, with years of protection and protection of the site as response variables.

**Fixed effects**	**Estimate (s. e.)**	***t***	***P***
**Intercept**	4.368 (0.395)	11.062	0.001
**Protection of the site**	-0.028 (0.368)	0.077	0.938
**Years of protection**	0.751 (0.187)	4.008	0.001
**Variance of random effects**			
**Residual**		11.29	
**Sampled spot nested in island**		9.69	
**Island**		0	

The best model for the biomass of potential Pacific harbor seal prey had the same variables, with none of the explanatory variables having a significant effect on the biomass of potential prey. In this model, it was the protection of the sample site that had a small negative effect on biomass (Tables 3 and S3).

**Table 3:** GLMMs of the fish biomass of Pacific harbor seal prey, with years of protection and protection of the site as response variables.

**Fixed effects**	**Estimate (s. e.)**	***t***	***P***
**Intercept**	3.511 (1.069)	3.284	**0.001**
**Protection of the site**	-0.624 (0.332)	1.881	0.059
**Years of protection**	0.665 (0.561)	1.185	0.236
**Variance of random effects**			
**Residual**		3.524	
**Sampled spot nested in island**		2.273	
**Island**		3.685	

### Biodiversity

**Fig 6:**
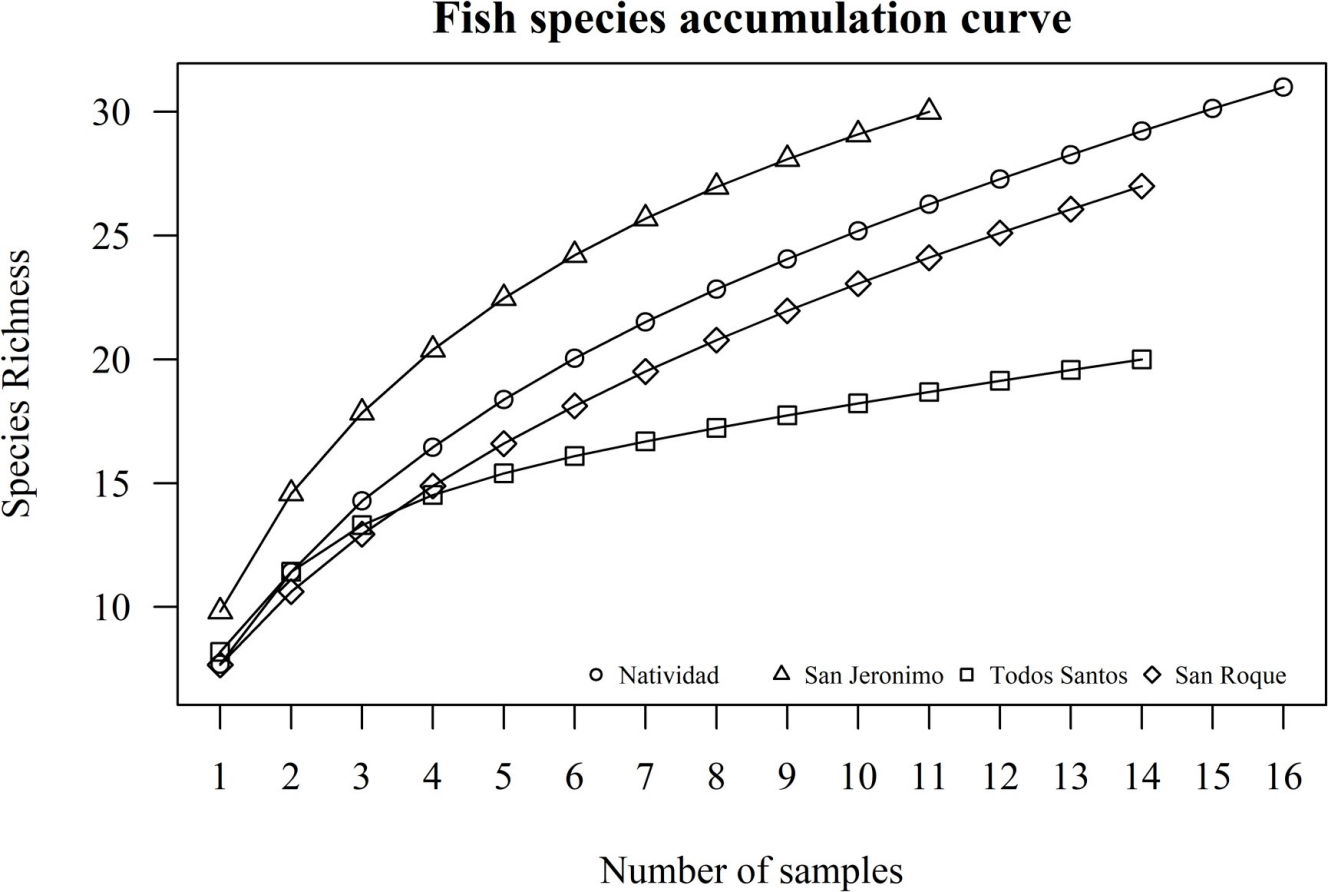
Fish species accumulation curve. The number of survey cylinders are represented on the X axis and the accumulation of new species recorded after taking survey data at each sample point is represented on the Y axis as species richness. Each line represents a different island.

Although fish species richness was higher around Natividad than the other islands (n = 31), the sampling effort here was also higher, as can be seen in the fish species accumulation curve (Fig 6). The effort around San Jeronimo was reduced due to weather conditions; however, considering the minimum common sampling effort, San Jeronimo presented the highest level of richness (n = 30). Fish diversity, which considers both richness and evenness, was significantly higher around Natividad than around the rest of the islands, according to Simpson’s index (F3,51 = 90.05, P < 0.001), followed by San Roque, San Jeronimo and Todos Santos, respectively.

**Fig 7:**
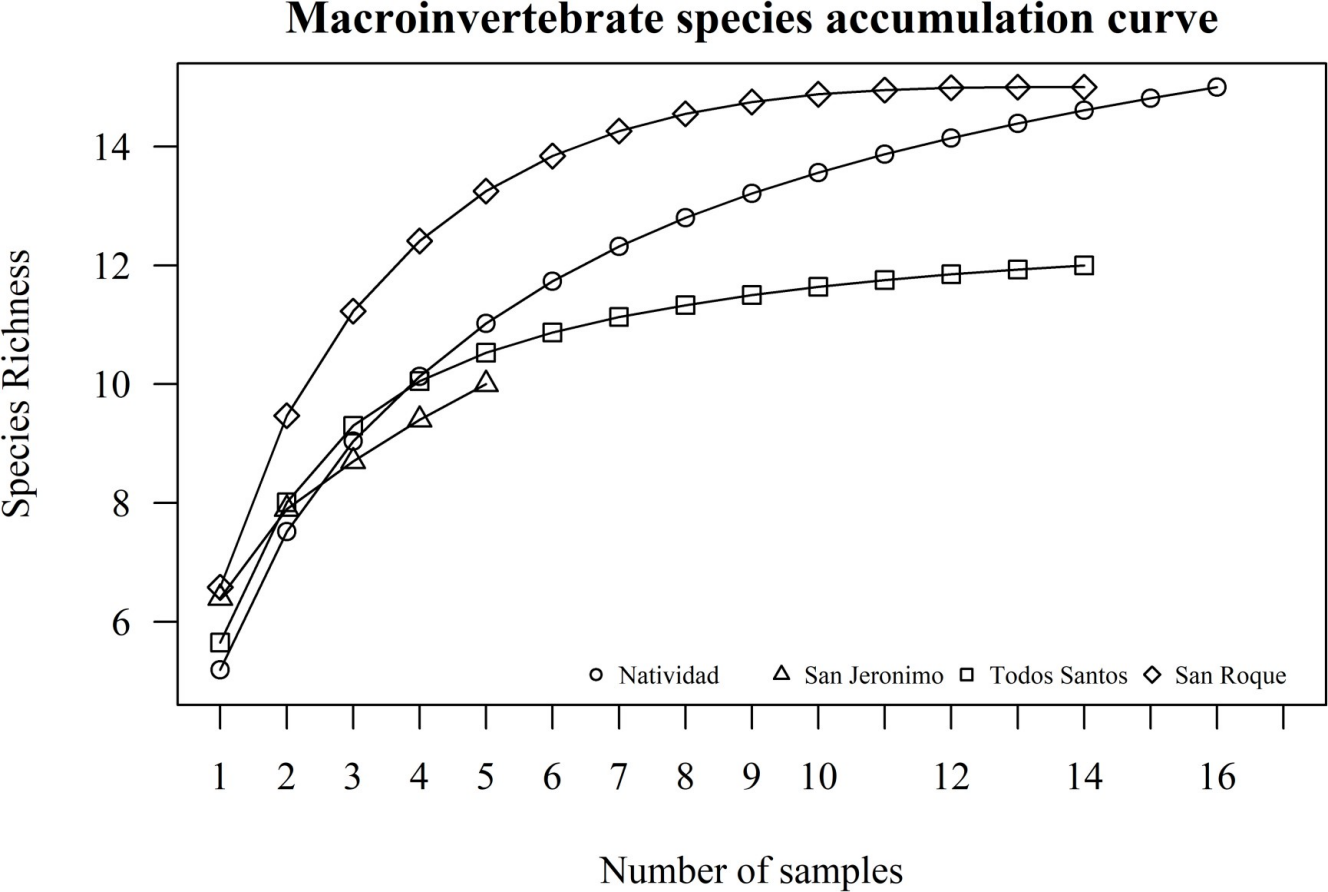
Macroinvertebrate species accumulation curve. The number of macroinvertebrate transects are represented on the X axis and the accumulation of new species recorded after taking survey data at each sample point is represented on the Y axis as species richness. Each line represents a different island. Only five transects were applied around San Jeronimo Island due to bad weather conditions.

Finally, the macroinvertebrate species accumulation curve (Fig 7) shows that Natividad presented the highest species richness value. Although the highest number of sampling points were applied on Natividad, richness plateaus were obtained around San Roque and Todos Santos islands before records were taken at the last sample point, meaning that sufficient sampling effort was undertaken. We were unable to estimate species richness around San Jerónimo Island due to adverse weather conditions. In terms of the Simpson diversity index, San Roque presented the highest mean (4.035, 95% CI = 3.817–4.13), followed by Todos Santos (3.515, 95% CI = 3.222–3.58) and Natividad (3.2, 95% CI = 3.079–3.217), with a significant difference found between at least in one of the means (Kruskal-Wallis, H = 23.98, DF = 2, P < 0.001). San Jerónimo was not included in the surveys for the Simpson diversity test due to the low number of survey sites.

## Discussion

The results of the present research showed that, even though similar exploitation methods and rates are found on all four islands studied here [7], fish biomass and diversity around Natividad Island is clearly superior to the other islands. According to our model, this may be attributed to the fact that marine reserves have been established around this island since 2006, given that the age of the reserve was a significant factor in the higher fish biomass found there. Moreover, the protection provided by the reserve was not significant, which may indicate a spillover effect, where biomass and diversity are similar both inside and outside these reserves. In other parts of the world, fish reserves have been demonstrated to increase the abundance and size of the fish inside them [2, 30, 31]. However, in order for them to be a successful management and conservation tool, it is necessary that there is a flow of individuals from the reserves to the surrounding fishing areas, which has proven difficult to verify [32]. In contrast, many studies have found increased abundance, over time, both inside marine reserves and in adjacent fishing areas over time [32–34]. The spillover success of different species depends on their characteristics, wherein they may be either density-dependent or independent because of random, migratory or behavioral movements [32].

In the case of San Jeronimo, the date on which the reserve was established was too close to the survey dates to show any significant improvements over islands without reserves. However, as with the results for Natividad, an effective increase both in biomass and in biodiversity may also be expected at San Jeronimo Island.

All four islands under study have important harbor seal colonies, with colony sizes of 473 individuals on Todos Santos, 523 on San Jeronimo, 724 on Natividad and 633 on San Roque recorded during the pupping season of 2009 [35]. Their diet on these islands consists mainly of benthic fish, such as California lizardfish (*Synodus lucioceps*), flatfish (*Citharichthys* spp.) rockfish (*Sebastes* spp.) and toadfish (*Porichthys notatus*), as well as octopus (*Octopus* spp.) and squid (*Doryteuthis opalescens*) [13]. When the marine reserves were established, local fishermen were concerned that pinniped foraging would prevent the recovery of target species in the reserves. However, we found that the biomass of potential harbor seal prey was higher around both Natividad and San Jeronimo compared to San Roque and Todos Santos, despite Natividad having the largest harbor seal rookery [36].

California sea lion colonies are typically larger than harbor seal colonies because their population in the region is much higher, estimated at 33,447 in 2010 [37], compared to 4,862 harbor seals in 2009 [38]. In 2010, the size of sea lion colonies on these islands was 449 on Todos Santos, 1,371 on San Jeronimo, and 1,176 on Natividad. While there are no sea lions on San Roque, 2,186 sea lions were recorded that year on Asuncion Island, 9.5 km to the southeast [39]. With little preference for specific prey, the California sea lion has sometimes been called an opportunistic predator [40]. However, a review of diet studies and databases in this region revealed the California sea lion to be a plastic specialist, meaning that it consumes many different resources in low quantities and few resources with a high frequency [12]. California sea lions consume more than 137 species of fish and 24 mollusk species, mainly cephalopods [12].

Our results showed that the biomass of potential California sea lion prey was higher around Natividad, with the model showing a significant effect of the age of the reserves, with Natividad the only island with eight-year-old reserves at the time of sampling. Around San Jeronimo, which has a colony with a similar size, the prey biomass mean was up to four times lower. This indicates that the implementation of marine reserves has not only the potential to recover fisheries production, but also to increase potential pinniped prey biomass and overall fish diversity, thus potentially benefiting pinniped populations [1, 9].

The last point discussed above is important in two main ways. Firstly, it would give these predators more resilience to cope with adverse oceanographic conditions, such as the El Niño-Southern Oscillation [38, 39], minimum oxygen zones, or other climate change effects, such as the movement of certain prey away from high-temperature water masses. The marine reserves around Natividad have already been proven to increase ecosystem resilience to minimum oxygen zones caused by climate effects [4]. Secondly, the above discussed effect has the potential to reduce conflicts between fishermen and pinnipeds, with severe conflicts occurring between them around the world, threatening both these marine mammals’ existence and the fisheries’ income [40]. There seems to be a trend, in that the more exploited a marine ecosystem is, the bigger the conflicts become [7, 41–44]. The fishermen of the cooperatives under study report conflicts with the California sea lion and, to a lesser extent, with the harbor seal populations. These animals tend to damage fishing gear, including lobster traps, and scare fish from the nets, although these conflicts are on a much lower scale than in other parts of the world. Comparing the four islands studied in the present research, Natividad had a lower percentage of complaints from fishermen about such conflicts than the other three [7].

In conclusion, our results show that overall fish biomass and diversity increases after marine reserves have been established for a number of years, even in the presence of large pinniped colonies. These reserves also have the potential to mitigate climate-induced stress on their populations, as well as increasing potential prey biomass; therefore, they may help pinniped population health and, by increasing prey availability, may reduce competition for common resources between fishermen and pinnipeds.

## Supporting information

S1 Table GLMMs of all fish biomass against years and protection of the site. (PDF)

S2 Table GLLMs using years of protection and protection of the site as response variables. (PDF)

S3 Table GLMMs of the fish biomass of all harbor seal prey against years and protection of the site. (PDF)
