# Supplementary material for: Do marine reserves increase prey for California sea lions and Pacific harbor seals?
Source: PLoS One. 2019 Jun 20;14(6):e0218651. doi: 10.1371/journal.pone.0218651 (PMC6586349; doi:10.1371/journal.pone.0218651)
Supplement: S1 Table — (PDF) [file pone.0218651.s001.pdf]

S1 Table. GLMMs of all fish biomass against years and protection of the site.

Fixed effects	AIC	Deviance	Variance of Residuals
**Anything**	114995	115025	12.573
**Years of protection**	114996	115034	12.571
**Years and protection of the site**	114997	115031	12.571
**Protection of the site**	114998	115040	12.573
**Years, protection and interaction**	114998	115049	12.569
