# Supplementary material for: Do marine reserves increase prey for California sea lions and Pacific harbor seals?
Source: PLoS One. 2019 Jun 20;14(6):e0218651. doi: 10.1371/journal.pone.0218651 (PMC6586349; doi:10.1371/journal.pone.0218651)
Supplement: S2 Table — (PDF) [file pone.0218651.s002.pdf]

S2 Table. GLLMs using years of protection and protection of the site as response variables.

**Fixed effects**	**Estimate (s. e.)**	***t***	***P***
**Intercept**	4.216 (0.650)	6.482	**0.001**
**Protection of the site**	-0.125 (0.318)	-0.393	0.694
**Years of protection**	0.716 (0.343)	2.089	**0.037**
**Variance of random effects**			
**Residual**	12.571		
**Sampled spot nested in island**	7.809		
**Island**	3.923		
