# Supplementary material for: Do marine reserves increase prey for California sea lions and Pacific harbor seals?
Source: PLoS One. 2019 Jun 20;14(6):e0218651. doi: 10.1371/journal.pone.0218651 (PMC6586349; doi:10.1371/journal.pone.0218651)
Supplement: S3 Table — (PDF) [file pone.0218651.s003.pdf]

S3 Table. GLMMs of the fish biomass of all harbor seal prey against years and protection of the site.

Fixed effects	AIC	Deviance	Variance of Residuals
**Anything**	62674.1	62666.1	3.526
**Protection of the site**	62673.0	62663.0	3.525
**Years and status of protection**	62674.0	62662.0	3.524
**Years, protection and interaction**	62675.0	62661.0	3.525

The ΔAIC between each model is below 2, making each model equally eligible.
